# Hidden in plain sight: Using administrative data to conduct a longitudinal cohort study of children exposed to opioids in pregnancy

**DOI:** 10.23889/ijpds.v8i2.2296

**Published:** 2023-09-14

**Authors:** Louise Marryat, Petra Rauchhaus, James P Boardman, Alison McFadden, Anne Whittaker, John Frank

**Affiliations:** 1 University of Dundee, Dundee, United Kingdom; 2 University of Edinburgh, Edinburgh, United Kingdom; 3 University of Stirling, Stirling, United Kingdom

## Objectives

Children of women who use substances are difficult to research at a population-level using traditional research methods due to the complexity of their lives. Resultingly, we have little robust evidence on their outcomes. This study developed an administrative data cohort of children exposed to opioids and explored health outcomes.

## Methods

Using data from birth records, antenatal records, prescription data, hospital/psychiatric hospital admissions, and drug and alcohol service data, we identified 6,408 children (born 2009-2019) in Scotland who were exposed to opioids through illicit use and/or medication assisted treatment (i.e. methadone/buprenorphine). A control group (n. 19,089) of children not exposed to opioids were matched on age of mother and Scottish Index of Multiple Deprivation. Data were described and linear and logistic regression models were used to examine the relationship between risk factors (such as drug and alcohol use in pregnancy, gestation at booking and at birth), and key early outcomes.

## Results

Although the majority of women had their substance use recorded in antenatal records, 28.9% did not, demonstrating the importance of using multiple administrative datasets to form the cohort. Children in the cohort were more likely to experience a range of adverse outcomes including being born early (17% born prematurely, compared with 6.5% in control group), having a below normal Apgar score (the scoring system used to assess newborns shortly after birth) (2.9% in cohort vs. 1.5% in controls), having significantly lower birthweight, length and head circumference, and more likely to be removed from their mother prior hospital discharge. Differences between the cohorts remained after controlling for other risk factors including alcohol use, and gestation.

## Conclusion

This feasibility study brought together a cohort of children usually excluded from traditional forms of research. The research demonstrated early differences in outcomes between exposed children and others from similar socio-economic groups. The next stage of this research is exploring health and development outcomes in the preschool period.

